# The "Llandovery Castle" Outrage

**Published:** 1918-07-13

**Authors:** 


### THE "LLANDOVERY CASTLE" OUTRAGE.

### Survivor's Story to the King.

The King heard on Thursday the story of the sinking of the hospital ship *Llandovery Castle* from one of the survivors—Sergeant Arthur Knight, of the Canadian Army Medical Corps, who was summoned to Buckingham Palace. Sergeant Knight was closely questioned by the King, especially as to what happened after the vessel was torpedoed, and His Majesty expressed his intense horror of the German outrage.

The conversation was private, but Sergeant Knight repeated his story to a Press representative afterwards. He told how after the torpedo had struck the ship he was able to get into a small boat with a crew of nine and fourteen of the nursing sisters. The boat was capsized and sucked under by the sinking ship, and he saw it no more unless it was a boat floating bottom upwards that he saw later. All the clothing he had been able to secure was a pyjama jacket. "I managed," he said, "to get hold of a piece of timber and floated about for what seemed to me quite two hours. Presently a boat came alongside my raft, an oar was put out to me, and I was pulled on board. It was then pitch dark. I heard an order in good but slightly accented English to go alongside what proved to be a submarine, and also heard a reply that we were trying to save others. The answer was, 'If you don't come alongside at once you will find my big gun at you.' On coming with a jar against the side of the submarine I grabbed hold of some projection and scrambled on board her. Seven or eight young Germans gathered round me and asked in good English, 'What do you want?' but without waiting for my reply, and after an exclamation which I did not hear accurately, four of them seized me and pitched me bodily back into the boat. There were, as I afterwards found, twenty-four of us in the boat, and we were brusquely ordered to 'get away quick.' We obeyed, but the submarine came after us, and seemed to be trying to 'down' us with her wash. We saw the submarine rushing about among the wreckage apparently trying to ram any other boat that might be afloat. Our boat got away, but for the next fifteen minutes we heard heavy reports and some shells passed screaming over us. I counted twenty shots, and concluded that survivors in the water were being fired upon. We pulled all night and all the next day, and were finally picked up by the *Lysander* at about 9.30 on Saturday morning."—*The Times*.

